# Complete Genome Sequences of an H5N1 Highly Pathogenic Avian Influenza Virus Isolated from Pigeon in China in 2012

**DOI:** 10.1128/genomeA.01330-15

**Published:** 2015-11-12

**Authors:** Yanfeng Yao, Shulin Fu, Bin He, Xiabing Chen, Zhiyong Shao, Wenhai Yang, Jie Chen

**Affiliations:** a Wuhan Institute of Animal Husbandry and Veterinary Science, Wuhan Academy of Agricultural Science and Technology, Wuhan, Hubei, People’s Republic of China; b College of Veterinary Medicine, China Agricultural University, Beijing, People’s Republic of China

## Abstract

An avian influenza virus strain, A/pigeon/Hubei/RP25/2012 (H5N1), was isolated from pigeons in Hubei province, China. Phylogenetic analysis indicates that the HA gene belongs to clade 2.3.4 and the other internal genes present different recombination events. Information about the strain provides basic research data for epidemiological evidences for revealing influenza evolution.

## GENOME ANNOUNCEMENT

Since its emergence in 1996, the H5N1 highly pathogenic avian influenza virus (HPAIV) has caused both poultry outbreaks and human infections in multiple countries, including China (1). Most human cases of the H5N1 associate with avian influenza virus (AIV)-infected poultry (2). Pigeons in urban areas live close to human activities; therefore, surveillance for H5 subtype AIV in pigeon is important.

In this study, we collected 30 cloacal swabs samples from apparently healthy pigeon in the Hubei province of central China. An H5N1 strain, named A/pigeon/Hubei/RP25/2012 (RP25), was isolated. The whole genome of this virus strain was amplified by reverse transcription-PCR (RT-PCR) using universal primers (3). The PCR products were purified, and sequenced (Invitrogen, Shanghai, China). The complete genome consists of 8 single-stranded RNA segments, PB2, PB1, PA, HA, NP, NA, M, and NS with 2,280, 2,274, 2,151, 1,704, 1,497, 1,350, 1,007, and 875 nucleotides, respectively.

Based on the deduced amino acid sequence of the HA gene, RP25 has a multibasic protease cleavage site sequence of RRRKR/G, which indicates high pathogenicity in chicken (4). At the receptor binding site in HA1, RP25 contains Q226 and G228, indicating an AIV receptor preference (H3 numbering) (5). Analysis of potential glycosylation sites of surface proteins revealed 7 potential N-linked glycosylation sites in HA (positions 27, 39, 170, 181, 302, 499, and 558) and 3 potential N-linked glycosylation sites in NA (positions 88, 146, and 235). There are 20 amino acid deletions at the NA stalk region and 5 amino acid deletions in the NS1 gene. The absence of His274Tyr mutation in the RP25 NA protein indicates that RP25 may be sensitive to neuraminidase inhibitors (6). The amino acids at residues 627 and 701 in the PB2 protein were glutamic acid (E), and aspartic acid (D), respectively, a characteristic of avian replication preference (7, 8).

Sequence analysis revealed that the nucleotide sequences of the HA gene of the RP25 strain shares 98% homology with that of a Hong Kong strain, A/Peregrine falcon/HongKong/810/2009 (H5N1), while the NA gene sequence is most closely related to that of A/duck/Liaoning/Q1/2009 (H5N1). The M gene has the greatest sequence identities (99%) with an H9N2 virus (A/swine/Yangzhou/1/2008). Other internal genes of RP25 are found to be more similar to those of southern China H5N1 AIV strains (97 to 99% nucleotide identity). Phylogenetic tree analysis indicates that the HA gene belongs to clade 2.3.4 and that other genes present different recombination features, which suggests that this H5N1 strain went through extensive evolution and recombination with different subtypes of influenza viruses.

In conclusion, RP25 is a novel reassortant avian influenza virus, and poses a potential threat to human. These results highlight the importance of persistent surveillance for H5 subtype AIV in birds.

### Nucleotide sequence accession numbers.

The complete genome sequence of A/pigeon/Hubei/RP25/2012 (H5N1) is available in GenBank under accession numbers KT587283 to KT587290.
